# The Human Cytomegalovirus Chemokine vCXCL-1 Modulates Normal Dissemination Kinetics of Murine Cytomegalovirus *In Vivo*

**DOI:** 10.1128/mBio.01289-19

**Published:** 2019-06-25

**Authors:** Joseph W. Jackson, Trevor J. Hancock, Ellen LaPrade, Pranay Dogra, Eric R. Gann, Thomas J. Masi, Ravichandran Panchanathan, William E. Miller, Steven W. Wilhelm, Tim E. Sparer

**Affiliations:** a Department of Microbiology, University of Tennessee, Knoxville, Tennessee, USA; b Columbia Center for Translational Immunology, Columbia University, New York, New York, USA; c Department of Molecular Genetics, University of Cincinnati College of Medicine, Cincinnati, Ohio, USA; d University of Tennessee Graduate School of Medicine, Department of Surgery, University of Tennessee Medical Center, Knoxville, Tennessee, USA; Princeton University; La Jolla Institute for Immunology; Oregon Health & Science University

**Keywords:** betaherpesvirus, neutrophils, vCXCL-1, viral chemokines, cytomegalovirus, MCMV

## Abstract

Human cytomegalovirus (HCMV) is a betaherpesvirus that is a significant pathogen within newborn and immunocompromised populations. Morbidity associated with HCMV infection is the consequence of viral dissemination. HCMV has evolved to manipulate the host immune system to enhance viral dissemination and ensure long-term survival within the host. The immunomodulatory protein vCXCL-1, a viral chemokine functioning primarily through the CXCR2 chemokine receptor, is hypothesized to attract CXCR2^+^ neutrophils to infection sites, aiding viral dissemination. Neutrophils harbor HCMV *in vivo*; however, the interaction between vCXCL-1 and the neutrophil has not been evaluated *in vivo*. Using the mouse model and mouse cytomegalovirus (MCMV) infection, we show that murine neutrophils harbor and transfer infectious MCMV and that virus replication initiates within this cell type. Utilizing recombinant MCMVs expressing vCXCL-1 from the HCMV strain (Toledo), we demonstrated that vCXCL-1 significantly enhances MCMV dissemination kinetics. Through cellular depletion experiments, we observe that neutrophils impact dissemination but that overall dissemination is largely neutrophil independent. This work adds neutrophils to the list of innate cells (i.e., dendritic and macrophages/monocytes) that contribute to MCMV dissemination but refutes the hypothesis that neutrophils are the primary cell responding to vCXCL-1.

## Abstract (precis)

An adequate *in vivo* analysis of HCMV’s viral chemokine vCXCL-1 has been lacking. Here we generate recombinant MCMVs expressing vCXCL-1 to study vCXCL-1 function *in vivo* using MCMV as a surrogate. We demonstrate that vCXCL-1 increases MCMV dissemination kinetics for both primary and secondary dissemination. Additionally, we provide evidence, that the murine neutrophil is largely a bystander in the mouse’s response to vCXCL-1. We confirm the hypothesis that vCXCL-1 is a HCMV virulence factor. Infection of severely immunocompromised mice with MCMVs expressing vCXCL-1 was lethal in more than 50% of infected animals, while all animals infected with parental virus survived during a 12-day period. This work provides needed insights into vCXCL-1 function *in vivo*.

## INTRODUCTION

Human cytomegalovirus (HCMV) is a serious pathogen in immunocompromised populations (1, 2) and the leading cause of infectious congenital disease (3, 4). Following *in utero* infection, fetal abnormalities such as microcephaly or other sequelae (e.g., progressive deafness and learning disabilities) can occur (5, 6). Primary infection or latent viral reactivation in immunocompromised adults (7, 8), such as cancer therapy patients, organ transplant recipients, or HIV/AIDS patients, can cause gastroenteritis, retinitis, or organ transplant rejection (2, 9). Regardless of the host, disease due to viral infection results from viral dissemination (10). Interestingly, HCMV has evolved numerous immunomodulatory proteins that blunt normal protective immune responses, restructure inflammatory environments, and ensure long-term survival in the host (11–13). The viral chemokine, vCXCL-1, is an HCMV protein that preferentially recruits CXCR2^+^ neutrophils over other innate immune cells *in vitro* (14–16).

In addition to engaging CXCR2 (14, 15), vCXCL-1 may also signal through human CXCR1 and CX3CR1 (15, 16). CX3CR1^+^/CXCR1^+^ natural killer (NK) cells functionally respond to vCXCL-1 albeit at a significantly reduced level compared to CXCR2^+^/CXCR1^+^ neutrophils (16). Unfortunately, any *in vivo* evaluation of the interaction of vCXCL-1 with the immune system and its contribution to CMV pathogenesis has been complicated by the species specificity of CMV (17–19).

Mouse cytomegalovirus (MCMV) infection of mice is frequently used to study CMV dissemination. MCMV has similar pathogenesis to HCMV, contains many homologues and orthologues to HCMV genes, and disseminates via innate immune cells (20, 21). MCMV does not encode vCXCL-1 but rather encodes a C-C chemokine, MCK2, that enhances dissemination (22–24). We have previously expressed vCXCL-1 from chimpanzee CMV in MCMV and observed no salivary gland dissemination (25, 26), potentially due to inappropriate timing and expression levels of the vCXCL-1 insert. Here, we engineered human vCXCL-1 from the HCMV Toledo strain (vCXCL-1_Tol_) with a 2A peptide linked to the MCMV chemokine MCK2 to ensure appropriate timing and expression levels. We employed the MCMV bacmid of the Smith strain (pSM3fr-MCK2-2fl) in which MCK2 aids viral entry in addition to its role in dissemination (24). Because of MCK2's dual role, we chose to fuse vCXCL-1_Tol_ to MCK2 instead of deleting it from the recombinant virus. We demonstrate that murine neutrophils are capable of harboring, transferring, and initiating MCMV replication and that CXCR2 stimulation is sufficient to alter MCMV dissemination kinetics. Additionally, we show that infections with recombinant MCMVs expressing vCXCL-1_Tol_ exhibit increased viral dissemination and virulence.

## RESULTS

### Murine neutrophils harbor, transfer, and initiate MCMV replication.

**FIG 1:**
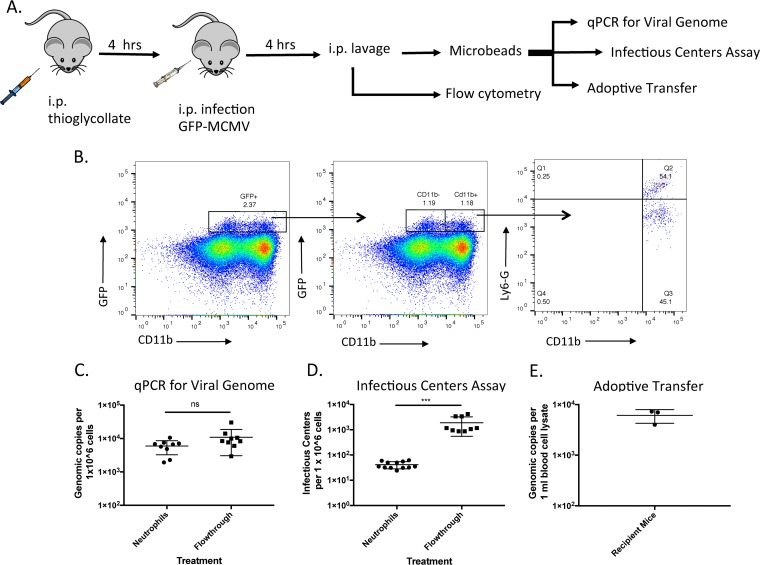
Neutrophils harbor and transfer MCMV *ex viv*o and *in vivo*. (A) Experimental design. (B) PECs were evaluated for the presence of GFP-expressing virus. Live GFP-expressing cells were gated for CD11b^+^ cells followed by Ly6-G^+^ gating. (C) Total DNA was extracted from 1 × 10^6^ purified neutrophils or Flowthrough cells. qPCR was performed to evaluate the number of MCMV genomes present. (D) Infectious center assay for determining the number of infected neutrophils. A total of 1 × 10^6^ purified neutrophils were incubated with an uninfected fibroblast monolayer for 5 days, and the number of plaques was evaluated. (E) MCMV genomes were quantified from whole blood isolated from mice that were adoptively transferred 1 × 10^6^ neutrophils from infected mice. Values are average titers ± standard deviations (SD) (error bars). Statistical significance was determined by Student’s *t* test and indicated as follows: ns, not significant; ***, *P* ≤ 0.001.

The capacity of neutrophils to impact cytomegalovirus dissemination is an area of contention. In blood of immunosuppressed patients, neutrophils harbor the largest viral burden (27, 28) and transfer infectious HCMV *ex vivo* and *in vitro*, but they cannot support productive viral replication (29, 30). The interaction between murine neutrophils and MCMV has not been evaluated. Here we use a thioglycolate inflammation model (31) to study the relationship between MCMV and murine neutrophils (Fig. 1A). Mice were infected with an MCMV encoding green fluorescent protein (GFP) under the CMV immediate early (IE) promoter (i.e., 4503) (22), and 4 h postinfection (Fig. 1B), total peritoneal exudate was harvested and analyzed by flow cytometry. Peritoneal exudate cells (PECs) in which virus has entered, uncoated, and expressed IE genes were expected to become GFP positive (GFP^+^). All live GFP^+^ cells were initially gated and further analyzed for the presence of neutrophil (Ly6G) and myeloid/granulocyte (CD11b) markers (Fig. 1B). Approximately 2% of all PECs were GFP^+^, with roughly half this population being CD11b^+^. Further analysis of the CD11b^+^ subpopulation indicated that ∼55% of these cells were Ly6G^+^ CD11b^+^ neutrophils and ∼45% were Ly6G^−^ CD11b^+^, likely dendritic cells or patrolling (resident) monocytes (32). The GFP^+^ CD11b^−^ PECs could be susceptible cell types such as epithelial and fibroblastic cells but not T or B cells (33). The gating strategy used to obtain these results is outlined (see Fig. S1B in the supplemental material). To ensure that the GFP signal is from cells that were infected and expressing GFP and not due to passive uptake or endocytosis of GFP^+^ virions, we infected cultured fibroblasts and used the translational inhibitor cycloheximide (34) to demonstrate that GFP expression is *de novo* and not due to passive uptake of GFP contamination in the viral preparation (Fig. S2).

10.1128/mBio.01289-19.1 FIG S1 Gating strategy used to identify GFP-positive cells. (A) Neutrophil (Ly6G^+^, CD11b^+^) purity following microbead isolation was evaluated with flow cytometry. Data are representative of two experiments with six or seven mice per experiment. Live cells were gated. (B) Following identification of live cells, single cells were analyzed for GFP expression. Gates were set on unstained infected peritoneal exudate cells. Download FIG S1, TIF file, 2.9 MB. Copyright © 2019 Jackson et al. 2019 Jackson et al. This content is distributed under the terms of the Creative Commons Attribution 4.0 International license.

10.1128/mBio.01289-19.2 FIG S2 *De novo* GFP expression. MEF 10.1 cells were infected with RM4503 at an MOI of 3 at 4 hours postinfection. GFP expression was determined via flow cytometry. (A to C) Uninfected cells (A), infected cells treated with 50 μg/ml of cycloheximide (CHX) for 1 hour prior to infection and 4 hours postinfection (B), or infected but untreated cells (vehicle control) (C). Download FIG S2, TIF file, 1.9 MB. Copyright © 2019 Jackson et al. 2019 Jackson et al. This content is distributed under the terms of the Creative Commons Attribution 4.0 International license.

We further evaluated the contribution of neutrophils to MCMV infection. Using anti-Ly6G microbeads, a 97% pure neutrophil population was prepared from peritoneal exudate from thioglycolate-treated, MCMV-infected mice (Fig. S1A). This population included approximately 25% of the total GFP^+^ cells as described above. The remaining population of peritoneal exudate cells (designated Flowthrough) represented ∼75% of all the GFP^+^ cells. When both populations were assayed for viral genome via quantitative PCR (qPCR) (Fig. 1C), we did not observe a significant difference in viral genome content between the two populations, indicating that neutrophils and other cell populations harbor MCMV genome 4 h postinfection (p.i.). As neutrophils are phagocytes and may be in the process of destroying virions, we carried out an infectious center assay. Consistent with this notion, the Ly6G^+^ population had dramatically less infectious MCMV compared with Flowthrough (Fig. 1D). To determine whether this neutrophil population was capable of infecting a new host, isolated neutrophils were adoptively transferred into uninfected immunocompetent mice. Three days after neutrophil transfer, blood was harvested and assayed for MCMV genome via qPCR. Transfer of infected neutrophils was sufficient to infect a new host (Fig. 1E), although it is possible that a contaminating cell population could contribute this infectivity as well.

### CXCR2 stimulation alters normal MCMV dissemination kinetics.

**FIG 2:**
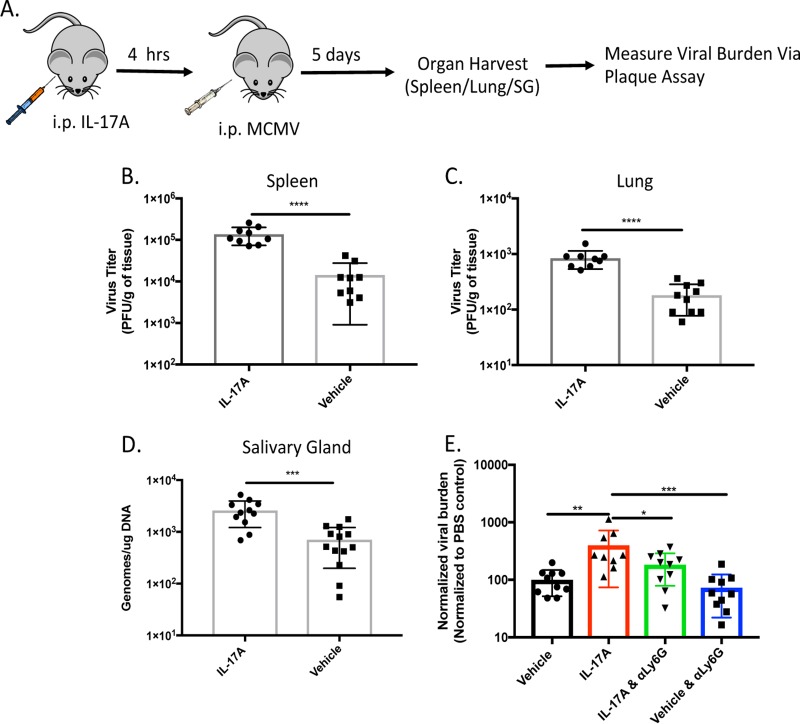
CXCR2 stimulation increases MCMV dissemination. (A) MCMV was administered i.p. during peak neutrophil response induced with IL-17A or vehicle control (not treated). (B and C) Viral titers of whole-organ homogenates were determined in the spleen (B) and lungs (C) of mice treated with IL-17A 5 days postinfection. *n* = 3 with 4 or 5 mice per group. (D) The salivary glands (SGs) of mice were harvested 5 days postinfection (dpi). Total DNA was harvested and subjected to qPCR for total MCMV burden in the SG. (E) Depletion of neutrophils reduces IL-17A-enhanced MCMV dissemination to the spleen. Plaque assays of spleens of mice 5 dpi. that received phosphate-buffered saline (PBS; i.e., vehicle), IL-17A alone, neutrophil-depleting antibody (anti-Ly6G [αLy6G]) coupled with IL-17A treatment (IL-17A & αLy6G), or neutrophil-depleting antibody alone prior to infection with MCMV. Data are from two pooled experiments. Data are normalized to the mean PFU/gram of tissue of the PBS control for each experiment. Values are average titers ± SD. Statistical significance was determined by Student’s *t* test or one-way ANOVA with Tukey’s multiple comparison of means and indicated as follows: *, *P* ≤ 0.05; **, *P* ≤ 0.01; ***, *P* ≤ 0.001; ****, *P* ≤ 0.0001.

Because vCXCL-1_Tol_ functions primarily through CXCR2 (14, 15, 35), we sought to understand whether a targeted CXCR2 response could alter normal MCMV dissemination kinetics. When mice are injected intraperitoneally (i.p.) with interleukin 17A (IL-17A), mesothelial cells release host CXCL-1 (i.e., Groα) eliciting a neutrophilic, CXCR2-mediated response (36). We confirmed reports that IL-17A treatment alters only the neutrophil population in the peritoneum 4 h following treatment (data not shown). We exploited this experimental setup to mimic the viral chemokine’s function *in vivo* and evaluate the contribution of the vCXCL-1/CXCR2 signaling axis on viral dissemination (Fig. 2A). Mice treated with IL-17A prior to i.p. infection with MCMV exhibited significantly greater viral burden in spleen and lungs 5 days postinfection (dpi) compared to control animals treated with vehicle control (Fig. 2B and C). IL-17A-treated animals also had greater levels of salivary gland (SG)-associated virus seeding 5 dpi (Fig. 2D). Although there was faster seeding, IL-17A-treated animals did not exhibit a significant difference in viral titers within this organ 14 dpi compared to control animals (data not shown).

Neutrophils were depleted 2 days prior to IL-17A treatment and MCMV infection, then treated every other day after infection with anti-Ly6G or vehicle control (Fig. S3A). The titers of the virus in the spleens were determined 5 dpi (Fig. 2E). Neutrophil depletion resulted in a decreased viral burden in the spleens of IL17A-treated mice, suggesting that in this inflammatory milieu, neutrophils play a role in dissemination perhaps in addition to normal lymphatic drainage from the peritoneum (37).

10.1128/mBio.01289-19.3 FIG S3 Confirmation of neutrophil depletion. Neutrophil depletion was evaluated by flow cytometry. (A) Four hours after IL-17A treatment, mice were euthanized, and PECs were harvested. PECs were assayed for the presence of neutrophils (Ly6G^+^, CD11b^+^). (B) Whole blood was assayed for the presence of neutrophils (Ly6G^+^, CD11b^+^) from neutrophil-depleted or vehicle control-treated mice prior to infection with recombinant MCMVs. Data are representative of two experiments with three animals per group. Download FIG S3, TIF file, 2.2 MB. Copyright © 2019 Jackson et al. 2019 Jackson et al. This content is distributed under the terms of the Creative Commons Attribution 4.0 International license.

### Generation of recombinant MCMV expressing vCXCL-1_Tol_.

**FIG 3:**
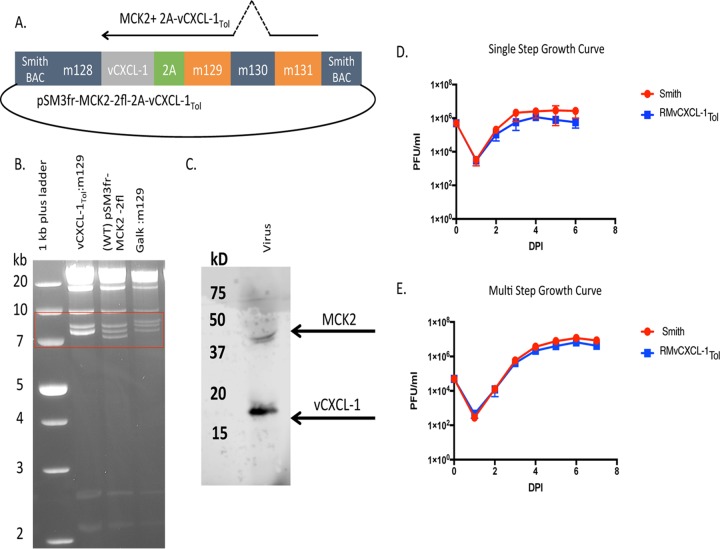
Generation of recombinant MCMV expressing vCXCL-1_Tol_. (A) Bacterial artificial chromosome (BAC) schematic of *vCXCL-1_Tol_* insertion into the *mck2* locus. (B) HindIII RFLP analysis of the BACs. The triplet banding patterns are boxed in red. (C) Western blot analysis of the viral supernatant of MCK2-2A-vCXCL-1_Tol_ MCMV. (D) Single-step growth curve (MOI of 5.0). DPI, day postinfection. (E) Multistep growth curves (MOI of 0.05). Values are average titers ± SD (error bars).

We have previously generated recombinant MCMVs that overexpress vCXCL-1 or host CXCL-1. These viruses disseminated normally to the spleen, liver, and lung but were defective in SG dissemination (25), leading to a hypothesis that levels and/or timing of chemokine expression hindered SG dissemination. Therefore, we engineered a recombinant MCMV in which vCXCL-1 from the HCMV Toledo strain (vCXCL-1_Tol_) was expressed as a cleavable fusion protein with the MCMV chemokine, MCK2. A picornavirus 2A self-cleaving peptide enabled the coexpression of the two chemokines (38) (Fig. 3A). The recombinant MCMV was generated by coupling classical bacterial recombineering with bacterial artificial chromosome from the Smith strain (pSM3fr-MCK2-2fl) (39) and CRISPR/Cas9 technology. HindIII RFLP analysis confirmed *galK* insertion into pSM3fr-MCK2-2fl, resulting in a 1.5-kb size increase of the 7.1-kb band into the top of the triplet band (Fig. 3B, boxed in red). The 500-bp addition of *vCXCL-1_Tol_* into the 7.1-kb band resulted in the creation of a doublet in the middle band of the HindIII triplet (Fig. 3B). The supernatants of pSM3fr-MCK2-2fl-infected MEF 10.1 cells were subjected to Western blot analysis using anti-FLAG antibodies to identify MCK2 and anti-HIS antibodies to identify vCXCL-1_Tol_ (Fig. 3C). Glycosylated MCK2 was detected at ∼40 kDa (40, 41) and vCXCL1 was detected at ∼17 kDa (26). These data indicate the self-cleaving 2A-peptide efficiently generated two independent proteins. In order to evaluate whether insertion of *vCXCL-1_Tol_* alters replication *in vitro*, both single-step and multistep growth curves were conducted (Fig. 3D and E). These analyses demonstrate that insertion of the *vCXCL-1_Tol_* gene does not alter viral replication *in vitro*.

### vCXCL-1_Tol_ expression alters normal MCMV dissemination kinetics.

**FIG 4:**
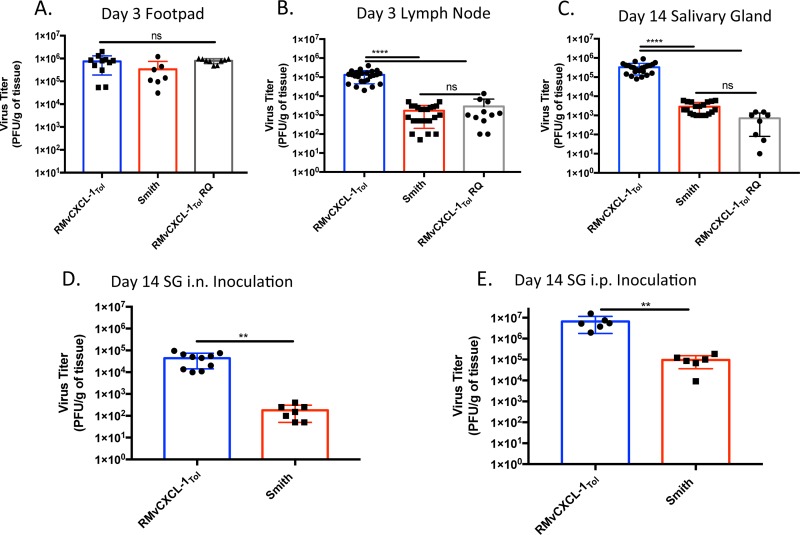
Infection of mice with vCXCL-1_Tol_-expressing MCMVs increases primary and secondary dissemination. Mice were infected by injecting 1 × 10^6^ PFU of either virus Smith, RMvCXCL-1_Tol_, or RMvCXCL-1_Tol_ RQ into their footpad (FP). (A to C) Plaque assays were performed on FP (A), lymph nodes (LN) (B), and salivary glands (SG) (C) that were harvested 3 (FP and LN) and 14 (SG) dpi. *n* = 3 to 5 experiments with at least three mice per group. Statistical significance was determined by one-way ANOVA with Tukey’s multiple comparison of the means. (D and E) Mice were infected either intranasally (i.n.) (D) or intraperitoneally (i.p.) (E), and plaque assays were performed on SG harvested 14 dpi. Results from two independent experiments with three or four mice per group are presented. Values are average titers ± SD (error bars). Statistical significance was determined by Student’s *t* test and indicated as follows: ns, not significant; **, *P* ≤ 0.01; ****, *P* ≤ 0.0001.

To test the hypothesis that vCXCL-1_Tol_ alters normal dissemination, we infected BALB/c mice with pSM3fr-MCK2-2fl expressing vCXCL-1_Tol_ (called RMvCXCL-1_Tol_) compared to parental pSM3fr-MCK2-2fl (Smith) and a rescued virus (called RMvCXCL-1_Tol_ RQ). RQ has had the MCK2-vCXCL1_Tol_ fusion replaced with the pSM3fr-MCK2-2fl *mck2* locus and serves as a control for adventitious mutations that could have arisen during recombineering. In addition, whole-genome sequencing was performed on recombinant MCMVs without revealing any unexpected mutations (data not shown). Even though mice inoculated in the footpad (FP) with recombinant MCMVs did not exhibit any difference in viral burden at the site of inoculation 3 dpi (Fig. 4A), RMvCXCL-1_Tol_ levels were ∼100-fold higher in the popliteal lymph node at this time compared to either pSM3fr-MCK2-2fl or RMvCXCL-1_Tol_ RQ (Fig. 4B). Therefore, the addition of vCXCL-1_Tol_ to MCMV does not alter replication at the infection site, but it does increase the levels of virus in the draining lymph node (LN). Importantly, vCXCL-1_Tol_ expression dramatically increased secondary dissemination to SG. Plaque assays of whole-organ homogenates revealed ∼100-fold increased viral levels in SG at 14 dpi compared to either wild-type (WT) or RQ viruses (Fig. 4C). These data indicate that vCXCL-1_Tol_ significantly increases both primary and secondary dissemination kinetics of MCK2-repaired pSM3fr bacmid-derived virus.

It has been reported that different routes of inoculation produce different dissemination patterns (32). To evaluate this possibility with our recombinant viruses, mice were inoculated intranasally (i.n.) (Fig. 4D) or i.p. (Fig. 4E) with RMvCXCL-1_Tol_ or Smith, and SGs were harvested for plaque assays at 14 dpi. Regardless of the inoculation route, expression of vCXCL-1_Tol_ enhances MCMV SG dissemination and/or replication.

### Analysis of cellular subsets responding to vCXCL-1_Tol_ *in vivo* early during infection.

**FIG 5:**
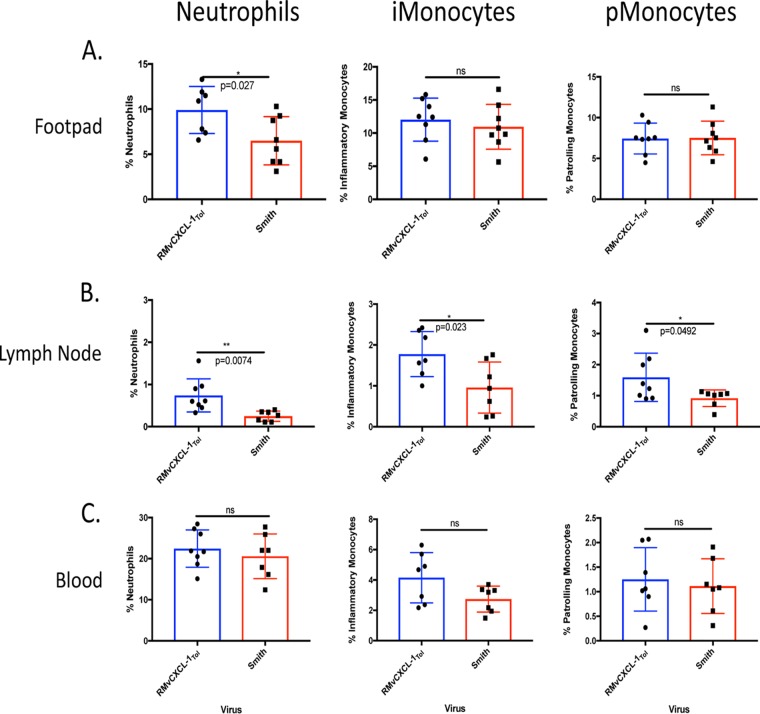
vCXCL-1_Tol_ alters cellular infiltrate in primary dissemination organs. Mice were infected via the footpads as in Fig. 4. (A to C) Footpads (A), lymph nodes (B), and peripheral blood (C) were harvested 3 dpi and evaluated by flow cytometry. Cells were characterized as follows: neutrophils, Ly6G^+^ CD11b^+^; inflammatory monocytes (iMonocytes), Ly6G^−^ Ly6C^hi^ CD11b^+^ CD11c^−^; patrolling monocytes (pMonocytes), Ly6G^−^ Ly6C^lo^ CD11b^+^ CD11c^+^. Values are average titers ± SD (error bars). Statistical significance was determined by Student’s *t* test and indicated as follows: ns, not significant; *, *P* ≤ 0.05; **, *P* ≤ 0.01.

Because vCXCL-1_Tol_ significantly increased dissemination independent of the inoculation site, we evaluated leukocytes recruited to the inoculation site, draining LN, and bloodstream following a FP infection early during infection to identify inflammatory cells potentially contributing to increased dissemination. Mice were infected as in Fig. 4, and FP, LN, and blood samples from infected animals were analyzed by flow cytometry at 3 dpi. In the FP (Fig. 5A), expression of vCXCL-1_Tol_ induced a significantly higher neutrophil influx compared to control virus, without altering the levels of either inflammatory monocytes (Ly6G^−^ Ly6C^hi^ CD11b^+^ CD11c^−^) or patrolling monocytes (Ly6G^−^ Ly6C^lo^ CD11b^+^ CD11c^+^) (32). Within the draining LN, all three leukocyte populations were significantly increased by vCXCL-1_Tol_ compared to control (Fig. 5B), while blood showed no difference (Fig. 5C). There was no difference in total leukocyte influx into the LN and blood (Fig. S4). These data suggest that vCXCL-1 recruitment of neutrophils to the infection site and draining LN or monocytes in LN could be responsible for increased dissemination to SG.

10.1128/mBio.01289-19.4 FIG S4 Total cell counts in LN and blood. At 3 days postinfection, LN (A) and blood (B) were harvested, and total leukocytes were counted. Blood was harvested via cardiac puncture and placed in lithium heparin-treated tubes. ACK lysis was performed to remove red blood cells, and leukocytes were counted. Download FIG S4, TIF file, 1.7 MB. Copyright © 2019 Jackson et al. 2019 Jackson et al. This content is distributed under the terms of the Creative Commons Attribution 4.0 International license.

### Removal of specific cellular subsets reveals insights into vCXCL-1_Tol_ function.

**FIG 6:**
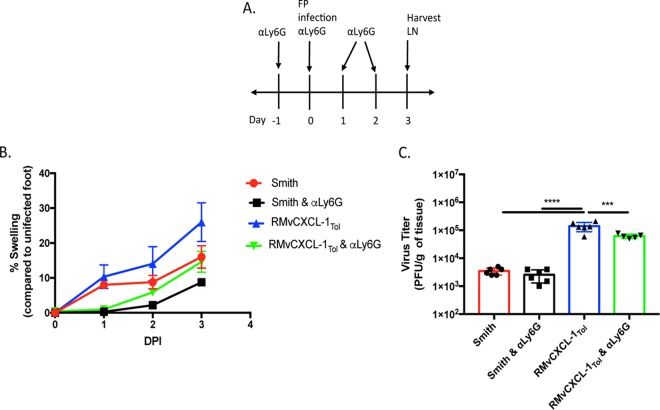
Neutrophils are partially responsible for vCXCL-1_Tol_-induced inflammation and dissemination. (A) Neutrophil depletion experimental design. αLy6G, anti-Ly6G antibody; FP, footpad; LN, lymph node. (B) Footpad swelling of infected footpads up to 3 dpi normalized to uninfected footpads. (C) Plaque assays were performed on LN harvested from depleted or control mice at 3 dpi. Values are averages of two experiments with three mice per group. Bars represent the average titers ± SD (error bars). Statistical significance was determined by one-way ANOVA with Tukey’s multiple comparison of means and indicated as follows: ***, *P* ≤ 0.001; ****, *P* ≤ 0.0001.

Using depleting antibodies, the importance of different cell types was tested. Neutrophil depletion with anti-Ly6G antibody prior to and during MCMV infection (Fig. 6A and Fig. S3B) reduced the level of vCXCL-1-induced swelling to levels observed with control virus in the absence of depletion (Fig. 6B). Neutrophil depletion also decreased to the level of swelling in controls. Thus, the increase in swelling due to vCXCL-1_Tol_ was dependent on neutrophils. To examine whether neutrophil depletion alters viral burden in different tissues, LNs were harvested from MCMV-infected mice that had either been neutrophil depleted or left untreated, and virus titers in the draining LN were determined 3 dpi (Fig. 6C). Neutrophil-depleted mice infected with RMvCXCL-1_Tol_ had less virus in the LN than non-neutrophil-depleted mice, but viral titers remained higher than the viral titers in the controls. These data indicate that neutrophils are partially responsible for vCXCL-1_Tol_-mediated dissemination, but they are not the major cell type responding to vCXCL-1_Tol_.

### vCXCL-1_Tol_ increases virulence.

**FIG 7:**
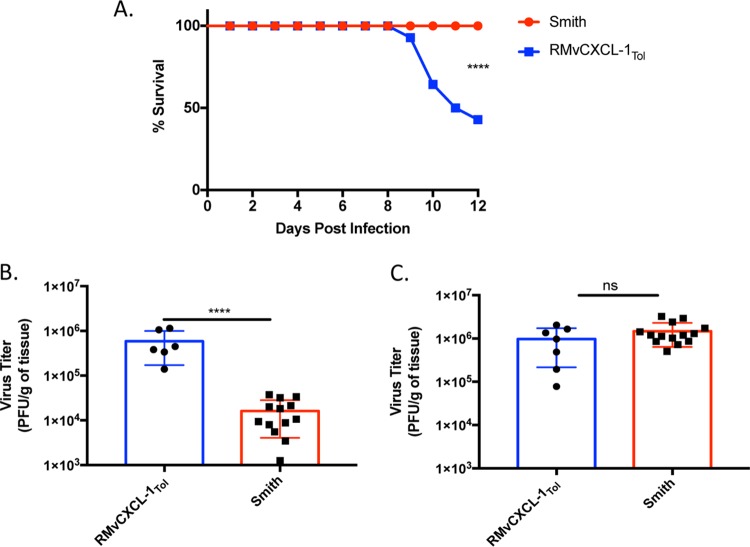
vCXCL-1_Tol_ increases virulence of MCMV in NSG mice which correlates with increased viral loads in the spleen. (A) Kaplan-Meier survival curve comparing NSG mice infected with either RMvCXCL-1_Tol_ or Smith MCMV. (B and C) At the termination of the experiment (12 days postinfection), MCMV titers in the spleen (B) or SG (C) were determined. Bars represent the average titers ± SD (error bars). Each symbol shows the viral titer for an individual mouse. Data are from three separate experiments with four to six mice per group for each virus. Statistical significance was ascertained using the Student’s *t* test and indicated as follows: ns, not significant; ****, *P* ≤ 0.0001.

Because there is no effective method for depleting monocytes without depleting other cellular subsets (42), we infected NOD−SCID−IL-2 gamma chain-deficient (NSG) mice. These mice lack T, B, and NK cells and have defective macrophages and dendritic cells (43). Although these mice are highly immunodeficient, monocytes and neutrophils remain functional. The number of mice surviving daily was recorded and plotted as a Kaplan-Meier survival curve (Fig. 7A). Interestingly, many mice infected with RMvCXCL-1_Tol_ died on or before 10 dpi, while all mice infected with the control virus survived to 12 dpi at which point SGs and spleens were harvested. These data indicate that vCXCL-1_Tol_ is a virulence factor, as 58% of mice infected with recombinant MCMVs expressing vCXCL-1_Tol_ died compared to mice infected with the Smith strain, which all survived. We also observed significantly higher viral titers in the spleens of mice during infection with RMvCXCL-1_Tol_ compared to the control group (Fig. 7B), even though there was no difference in SG viral titer for mice in both these groups at 12 dpi (Fig. 7C). These data point to either viral load in the spleen, inflammatory infiltrates into the liver (Fig. S5), or another unknown effect of vCXCL-1_Tol_ as a determinant for mortality in NSG mice.

10.1128/mBio.01289-19.5 FIG S5 vCXCL-1_Tol_ increases MCMV liver inflammation in NSG mice. (A) Liver samples from NSG animals harvested at day 12 postinfection were sectioned and stained with H&E using standard procedures. Representative sections from animals infected with strain Smith and RMvCXCL-1_Tol_ are shown. (B) Two independent sections of liver from each infected animal were counted using an Evos XL Core Microscope. The graph depicts the number of infiltrates quantified from each animal as well as an overall average from five or six animals in each group. Horizontal bars (± SD) represent the average infiltrate counts, and the numerical average is shown to the left of the bar. Each symbol represents the infiltrate count from an individual mouse. Download FIG S5, TIF file, 2.2 MB. Copyright © 2019 Jackson et al. 2019 Jackson et al. This content is distributed under the terms of the Creative Commons Attribution 4.0 International license.

## DISCUSSION

As most HCMV infections are asymptomatic, studying its dissemination from primary infection to the establishment of latency in humans has been limited to *in vitro* and *ex vivo* analysis (1, 2). These studies revealed that innate immune cells are major reservoirs of HCMV in blood and contribute to viral dissemination (28). HCMV’s reliance on innate immune cells has resulted in the evolution of immunomodulatory proteins such as chemokines that are capable of activating and recruiting innate immune cells (13). The discovery of the HCMV chemokine vCXCL-1 led to the hypothesis that vCXCL-1 recruits innate immune cells to the infection site and that these recruited cells contribute to HCMV dissemination (29, 44, 45). An adequate *in vivo* analysis of vCXCL-1 function is lacking. Here we employed a mouse model using recombinant MCMV expressing HCMV vCXCL-1 *in vivo*. A major limitation of the MCMV model and the reason recombinant MCMVs were generated is that some of the immunomodulatory proteins of MCMV and HCMV differ. MCMV encodes a C-C chemokine which also aids viral entry in MCMV Smith strain (24). However, HCMV encodes C-C and C-X-C chemokines (13), enhancing recruitment of multiple leukocyte subtypes (i.e., monocytes, neutrophils, and NK cells) *in vitro* (15, 16, 46). vCXCL-1 attracts neutrophils and potentially NK cells (15, 16). These observations suggest that neutrophils could be major players in HCMV dissemination.

To establish our model, we needed to understand MCMV-neutrophil interactions and how/whether neutrophils have a role in MCMV dissemination. This is important because neutrophils transfer HCMV (29), and vCXCL-1 alters human neutrophil functions (15). We show in a thioglycolate-induced model that murine neutrophils harbor and transfer MCMV to new cells although not as efficiently as other cells in the Flowthrough. This could be due to the neutrophil’s shorter half-life *ex vivo* or to the fact that neutrophils have phagocytosed viral genomes that are not infectious. We also show that viral transcription/translation begins in neutrophils (i.e., GFP expression) (Fig. 1), demonstrating that murine neutrophils can harbor MCMV.

Because vCXCL-1 is expressed late in HCMV’s life cycle (1), it is likely that infectious viral particles are being released simultaneously with chemokine expression. Therefore, we sought to mimic this environment. IL-17A injected i.p. into mice induces CXCL-1 (i.e., Groα) expression (36). CXCL-1 is a host chemokine that uses the CXCR2 receptor (47), inducing primarily a neutrophilic influx into the peritoneal cavity. Stimulating the CXCR2 signaling axis during infection increased viral burden in primary dissemination organs (i.e., spleen and lungs) and resulted in increased SG seeding. However, IL-17A treatment prior to infection did not lead to increased viral burden in SGs 14 dpi. This potentially could be due to a variety of factors, including localization or longevity of CXCR2 stimulation, as IL-17A treatment is transient. Therefore, it is not surprising that IL-17A treatment impacted only primary dissemination. Interestingly, when neutrophils were depleted from the IL-17A inflammation model, there was only a slight decrease in viral burden compared to nondepleted animals. This points to another CXCR2^+^ cell or another chemokine/cytokine-responsive cell responding to IL-17A. While neutrophils are the only statistically significant population that changes in response to IL-17A (36; data not shown), there could be a biologically relevant subset of CXCR2^+^ cells (48–50) responding to CXCL1 and aiding viral dissemination. It will be important to determine what cell types (e.g., inflammatory monocytes, NK cells, macrophages, etc.) are becoming infected immediately after IL-17A treatment.

Previously, we have demonstrated that overexpressing vCXCL-1 in the context of MCMV infection did not change primary dissemination but inhibited secondary dissemination (25). We speculated that the timing and quantity of viral chemokine expression induced an abnormal inflammatory environment, resulting in expedited or premature viral clearance. To alleviate this concern, we generated a recombinant MCMV expressing vCXCL-1 at relatively normal physiological times and levels by linking it to the MCMV protein MCK2. When mice were infected with recombinant vCXCL-1_Tol_ MCMV, there was a significant difference in viral dissemination kinetics for both primary and secondary dissemination compared to controls. Because different inoculation routes have different dissemination mechanisms, it was important to determine whether the phenotype observed was inoculation route dependent (32, 51). We showed that the RMvCXCL-1_Tol_ dissemination phenotype was still present whether mice were inoculated by the more natural inoculation route (i.e., intranasal [51]) or the more common intraperitoneal route. Because vCXCL-1_Tol_ alters neutrophil functions (15), murine neutrophils were depleted to dissect this relationship *in vivo*. As with IL-17A neutrophil depletion, neutrophil depletion coupled with recombinant viral infection significantly reduced viral dissemination but did not return viral burden to WT MCMV levels. These data indicate that there is another cellular target through which vCXCL-1_Tol_ is functioning.

As monocytes have also been shown to express CXCR2 under certain conditions (48, 52), the next logical experiment would be to deplete monocytes. Unfortunately, specific depletion of monocytes is not efficient (42). Instead, we chose to utilize the NSG mouse model. These mice are highly immunocompromised and lack mature B, T, and NK cells (43) and have defective macrophages and dendritic cells, which could participate in MCMV dissemination (32, 51, 53). However, NSG mice have functional monocytes. Because neutrophils were not the major cell type aiding dissemination (Fig. 6), we hypothesized that if viral dissemination of vCXCL-1_Tol_-expressing recombinant MCMV were still increased compared to WT MCMV in NSG mice, it would point toward a monocytic response to vCXCL-1_Tol_. Surprisingly, infection of NSG mice with RMvCXCL-1_Tol_ and not strain Smith resulted in death (Fig. 7). This was not expected, but these results support the claim that vCXCL-1_Tol_ is a virulence factor (12, 15, 26, 54). Additionally, Fig. 7 shows that primary dissemination (i.e., dissemination to the spleen) is significantly different between the two viruses, but there is no difference in secondary dissemination. This could indicate that primary dissemination and secondary dissemination are separate events mediated by different cell types, similar to our findings with CXCL-1-overexpressing MCMVs (25).

While neutrophils are not the major cell type aiding dissemination, our results provide evidence that murine neutrophils contribute to MCMV dissemination. We also report that vCXCL-1_Tol_ significantly enhances MCMV dissemination and pathogenesis. However, the hypothesis that neutrophils are the sole responders to vCXCL-1_Tol_ is incorrect. Other CXCR2-positive cells such as monocytes (48, 52) and myeloid-derived suppressor cells (55, 56) could be other targets for vCXCL-1. This hypothesis supports previous findings in which monocytes are major drivers of CMV dissemination (1, 2, 32, 57). Interestingly, the deaths of NSG mice infected with vCXCL-1_Tol_ MCMV provide supporting evidence that this protein is a virulence factor albeit in NSG mice. We have previously shown that vCXCL-1 from different HCMV strains vary in their ability to activate and induce neutrophil recruitment (15), but now we have a system to test their virulence *in vivo*.

## MATERIALS AND METHODS

### Plasmids.

pCas9 was a gift from Luciano Marraffini (Addgene plasmid 42876) (58). The selectable marker was replaced with kanamycin using Gibson Assembly. The p2A-Tol open reading frame (ORF) was synthesized (Genscript) after adding the P2A sequence (Addgene). This plasmid contains 250 bp of MCK2 that is FLAG tagged followed by the 2A peptide and vCXCL-1 from the HCMV Toledo strain, which is 6× HIS tagged. pcDNA-MCMV IE1 was generated as a qPCR standard.

### Cells and mice.

All experiments were performed with low-passage (<20) cells. Mouse embryonic fibroblast 10.1 (MEF 10.1) (59) were cultured in Dulbecco modified Eagle medium (DMEM) (Corning) supplemented with 10% Fetalclone III serum (HyClone, Logan, UT), 1% penicillin-streptomycin, and 1% l-glutamine.

BALB/c mice were purchased from Jackson Laboratory (Bar Harbor, ME) and housed under specific-pathogen-free conditions. Four- to 5-week-old nonobese diabetic severe combined immunodeficient, IL-2 common γ chain null (NSG) mice were purchased from Jackson Laboratory (Bar Harbor, ME). The Institutional Animal Care and Use Committees (IACUCs) at the University of Tennessee and University of Cincinnati approved all animal procedures.

### Viruses, BAC mutagenesis, and recombinant virus generation.

RM4503 virus was a gift from Ed Mocarski, Emory University (22), and MCMV Smith strain virus was derived from the pSM3fr-MCK2-2fl bacterial artificial chromosome (BAC) (originally from B. Adler) was a gift from Chris Benedict (60). MCMV was produced *in vitro* using MEF 10.1 cells. All viruses were stored at −80°C until use. Viral titer was assessed by plaque assay (described below) on MEF 10.1 cells.

BAC mutagenesis was performed on pSM3fr-MCK2-2fl BAC by coupling *galK* recombineering (39) with CRISPR/Cas9 technology. Briefly, Escherichia coli SW105 containing pSM3fr-MCK2-2fl was induced to express lambda red recombinase, and *galK* was inserted into the MCK2 locus, resulting in pSM3fr-MCK2-2fl-galK. SW105 cells containing pSM3fr-MCK2-2fl-galK were induced by heat and pCas9 with a gRNA targeting *galK* along with a PCR product containing the MCK2-2A-vCXCL-1_Tol_ DNA sequence was transformed into them. Transformants were plated on chloramphenicol and kanamycin. The following day, colonies were streaked onto MacConkey agar base (Difco) containing 1% galactose. Colonies that retained the *galK* gene were pink, while transformants with the desired recombination were white. SW105 cells containing recombinant BACs were further assessed via HindIII restriction fragment length polymorphism (RFLP) analysis, and the 2A-Tol insert was sequenced via Sanger sequencing after PCR amplification.

vCXCL-1_Tol_ and control BACs were transfected into MEF 10.1 cells using LT1 transfection reagent (Mirus). BAC origin excision was achieved through serial passage as previously described (61). Confirmation of loss of the BAC origin was assessed in purified viral particles instead of infected cells and then evaluated with PCR. Approximately 10 serial passages were needed to effectively excise the BAC origin.

### Western blotting.

Roller bottle (850 cm^2^) (Coring) supernatant from infected MEF 10.1 cells was harvested at 100% cytopathic effect (CPE). Ni-NTA beads were used to purify 6× His proteins (vCXCL-1_Tol_) and anti-FLAG agarose beads (Sigma) were used to purify FLAG-tagged proteins (MCK2) from approximately 60 ml of supernatant. Purified proteins were combined 1:1 with 6× His and FLAG. Proteins were run on a 15% SDS-PAGE and blotted onto an AZURE Biosystems membrane. Anti-His and anti-FLAG AZURE Western kits were used. Membranes were developed using an Odyssey Clx Li-Cor. Blots were analyzed using Image Studio v4.0.

### Sequencing and genome assembly.

Sequencing was conducted as previously described (62). Genome assembly was conducted with Geneious version 11.1.5. Briefly, purified viral DNA was harvested using quick -gDNA miniprep kit (Zymo Research). The sequencing was designed to ensure a 40× genome coverage. Illumina sequencing reads (150 × 150 paired-end sequencing reads) were mapped to the parental MCMV C5X reference genome (wild-type [WT] Smith strain). Geneious software’s SNP-finding function was used to find mutations and single nucleotide polymorphisms (SNPs). All resequencing data are available from authors upon request.

### qPCR MCMV quantification.

SYBR green real-time quantitative PCR (qPCR) was performed to measure viral load using primers designed to detect MCMV IE1 (63): IE1 Forward (5′-AGCCACCAACATTGACCACGCAC-3′) and IE1 Reverse (5′-GCCCCAACCAGGACACACAACTC-3′). Copy number was standardized using pcDNA-MCMV IE1. PCR was performed using a Chromo4 DNA engine PCR system (Bio-Rad). Quantification of viral DNA (IE1) was carried out using MJ Opticon Monitor analysis software version 3.1.

### Peritoneal inflammation models and neutrophil purification.

Thioglycolate-induced peritoneal inflammation was conducted as previously described (31). Briefly, mice were injected with 3% Brewer’s thioglycolate (BD). Four hours after the mice were injected with BD, the mice were infected with 1 × 10^6^ PFU of MCMV. Three hours later, the mice were euthanized, and peritoneal exudate cells (PECs) were harvested. Inflammation induced by IL-17A (Shenandoah Biotechnology Inc.) was performed as previously described (36).

Neutrophil purification was conducted using an anti-Ly6G microbead purification kit (Miltenyi Biotec). Briefly, 9 or 10 mice were administered 3% thioglycolate, and PECs were harvested 4 h postinjection. Peritoneal exudate was pooled from all mice, and the resulting single-cell suspension was subjected to microbead purification. Purity was determined by flow cytometry.

### Flow cytometry.

For FP infections, feet (cut at the ankles) were minced into small pieces (∼3 mm) and incubated on a rotatory shaker at 37**°**C for 1 h in a 0.5% (wt/vol) solution of type I collagenase (Worthington). The suspension of cells, LN, or PECs were passed through a 40-μm cell strainer (Fisher Scientific). Red blood cells were lysed with ACK (i.e., ammonium chloride-potassium) lysis buffer. Cells were stained for flow cytometry analysis with the following fluorochrome-conjugated antibodies for cellular subsets: anti-Ly6G (1A8), anti-Ly6C (HK1.4), anti-F4/80 (BM8), and anti-CD11c (N418) (all from BioLegend), anti-CD49b (DX5) from eBioscience, and anti-CD11b (M1/70) from BD Pharmingen. Data were acquired on BD LSR II flow cytometer (BD Biosciences) and analyzed using FlowJo software, version 10.1.

### Neutrophil depletion.

*In vivo* depletion of neutrophils was performed using 1A8 (anti-Ly6G) (BioXcell) as previously described (64). Briefly, depleting antibodies were administered every day starting at 2 days prior to MCMV infection or IL-17A treatment and then every other day until harvest. Neutrophils were depleted using 0.25 mg/inoculation with anti-Ly6G. Flow cytometry was used to confirm depletion.

### Plaque assay.

Plaque formation assay on MEF 10.1 cells was used to determine viral titers in organs. Briefly, MEF 10.1 cells were plated in a six-well dish. Organs were harvested and homogenized. Homogenate was serially diluted and added to MEF 10.1 cells and incubated for 1 h. After incubation, diluted virus was removed, and cells were overlaid with carboxylmethyl cellulose (CMC) medium and incubated for 5 days. CMC was removed, and plates were stained with Coomassie blue.

### Adoptive transfer of neutrophils.

Four hours after i.p. thioglycolate injection, 9 or 10 mice were infected i.p. with MCMV (RM4503) (22). Four hours postinfection, the mice were euthanized, and PECs were isolated. Neutrophils were purified using MACs beads as described above. Neutrophils (1 × 10^6^) were injected into the footpads of naive mice, and the amount of MCMV in the blood was quantified from 250 μl of whole blood via qPCR at 3 days after transfer.

### *In vitro* growth assay.

MEF 10.1 cells were plated in triplicate in a six-well dish and infected with Smith or RMvCXCL-1_Tol_ recombinants for either a multistep (multiplicity of infection [MOI] of 0.05) or single-step (MOI of 5) growth analysis. Supernatants were collected at the indicated times p.i. and sonicated prior to assessing the titer. The titers of viruses were determined via plaque assay.

### Infectious center assay.

The infectious center assay was performed as previously described (65). Briefly, PECs were harvested, and red blood cells were lysed with ACK lysis buffer. PECs or purified neutrophils (1 × 10^6^) were incubated for 12 h on an uninfected monolayer and overlaid with CMC medium. Plaques were counted after 5 days.

### Virulence studies.

NSG mice (four to six animals per virus strain per experiment) were infected i.p. with 5 × 10^5^ PFU of either RMvCXCL-1_Tol_ or Smith. Mice were monitored daily for weight loss and administered supportive care if necessary. Mice were euthanized at day 12, SG and spleens were removed, and virus titers in organs were determined by plaque assay.

### Statistical analysis.

Statistical significance was determined using Student’s *t* test or one-way analysis of variance (ANOVA) with Tukey’s multiple comparison of means using Prism 7 (GraphPad Software, Inc.).
