# Supplementary figures and images for: The Human Cytomegalovirus Chemokine vCXCL-1 Modulates Normal Dissemination Kinetics of Murine Cytomegalovirus In Vivo
Source: mBio. 2019 Jun 25;10(3):e01289-19. doi: 10.1128/mBio.01289-19 (PMC6593410; doi:10.1128/mBio.01289-19)

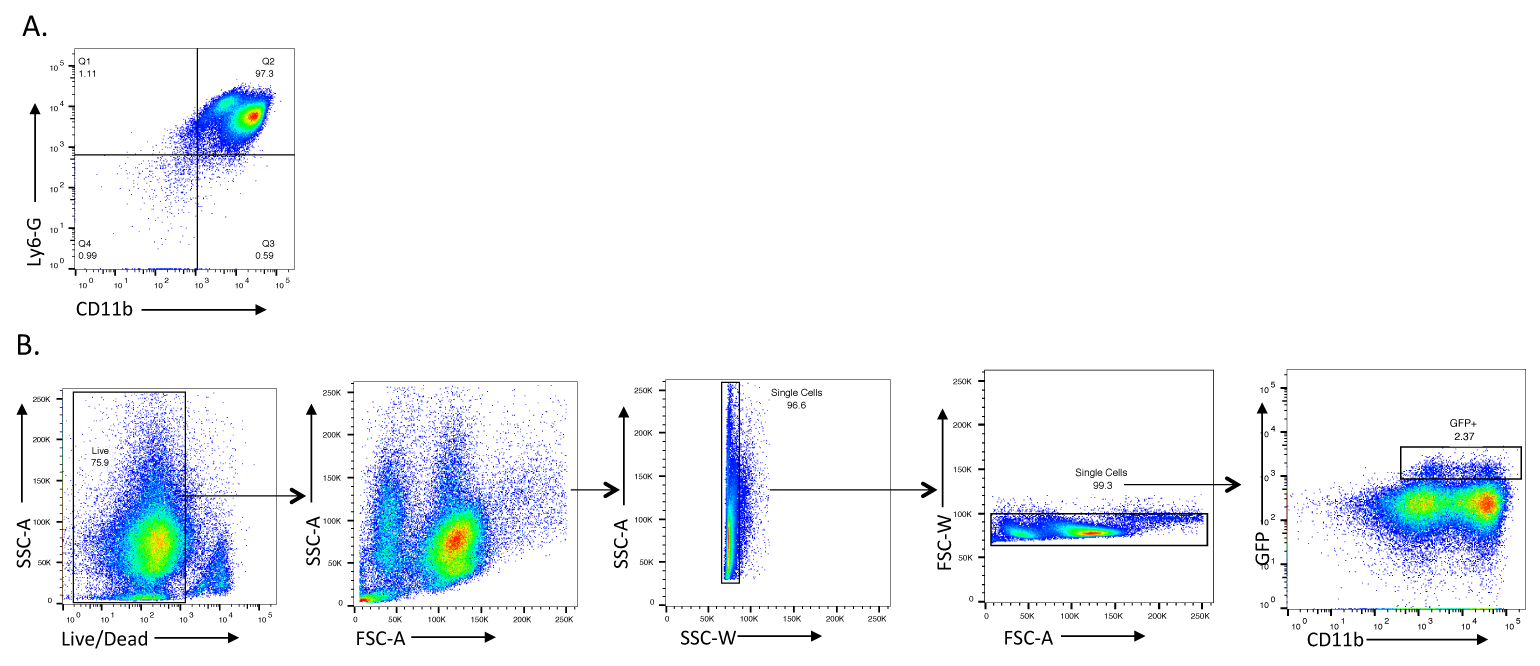

Supplement: FIG S1 [file mBio.01289-19-sf001.tif]

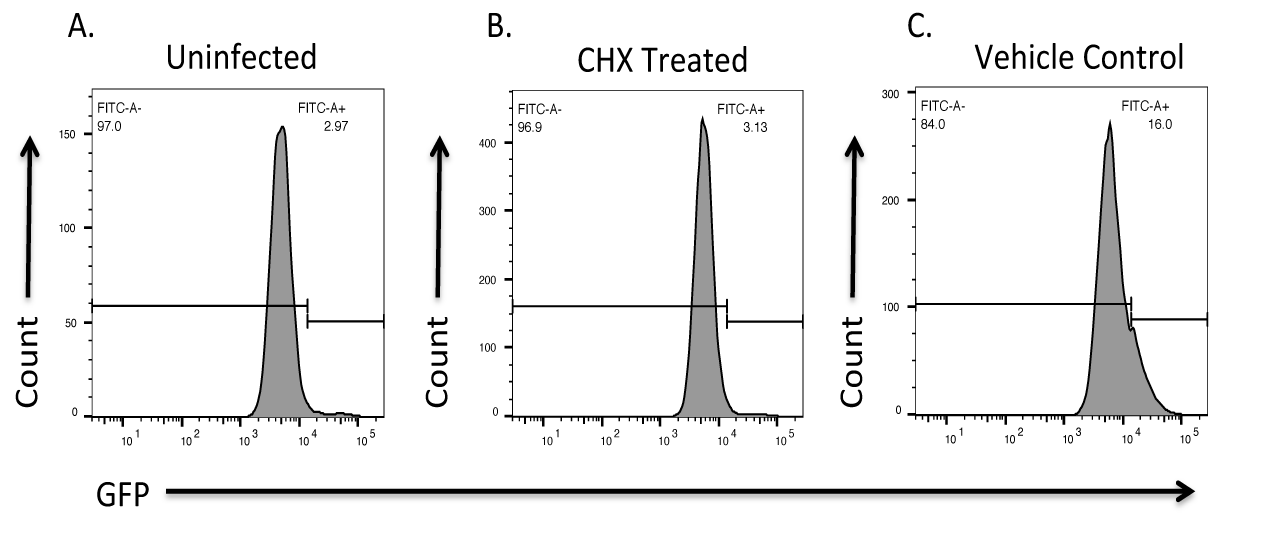

Supplement: FIG S2 [file mBio.01289-19-sf002.tif]

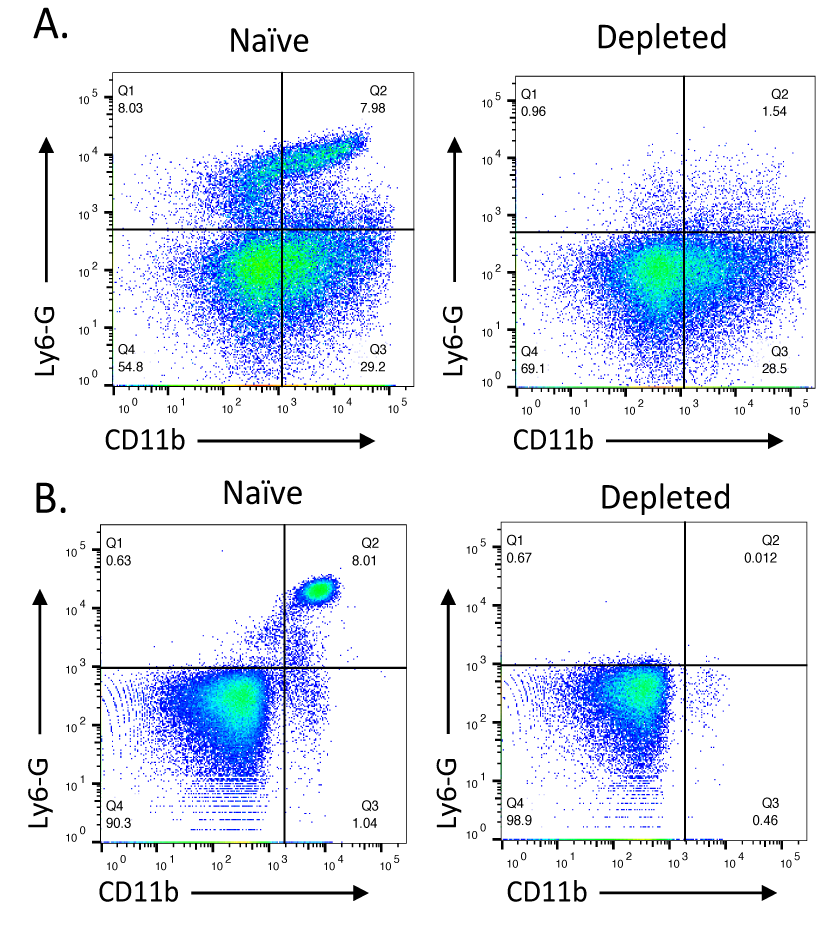

Supplement: FIG S3 [file mBio.01289-19-sf003.tif]

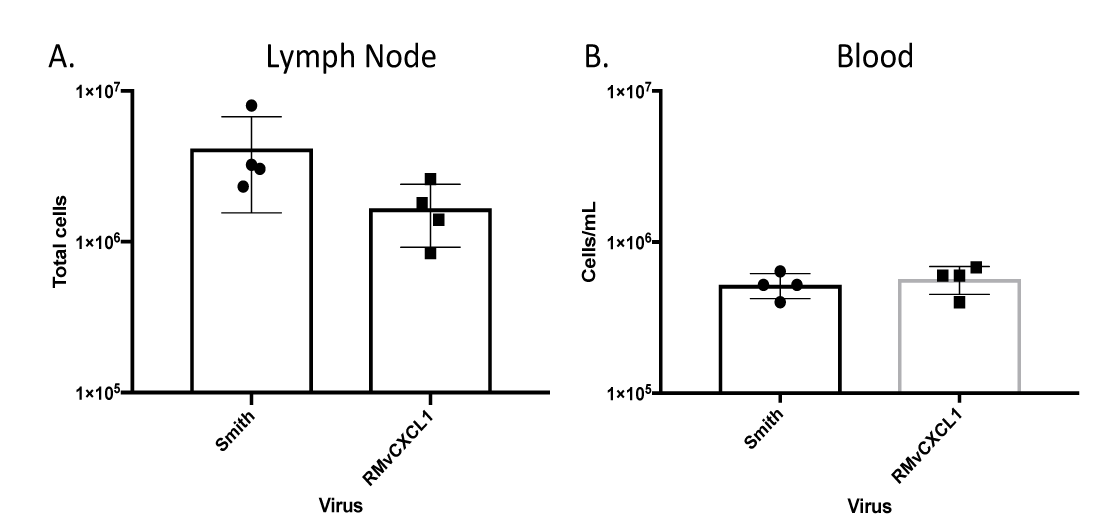

Supplement: FIG S4 [file mBio.01289-19-sf004.tif]

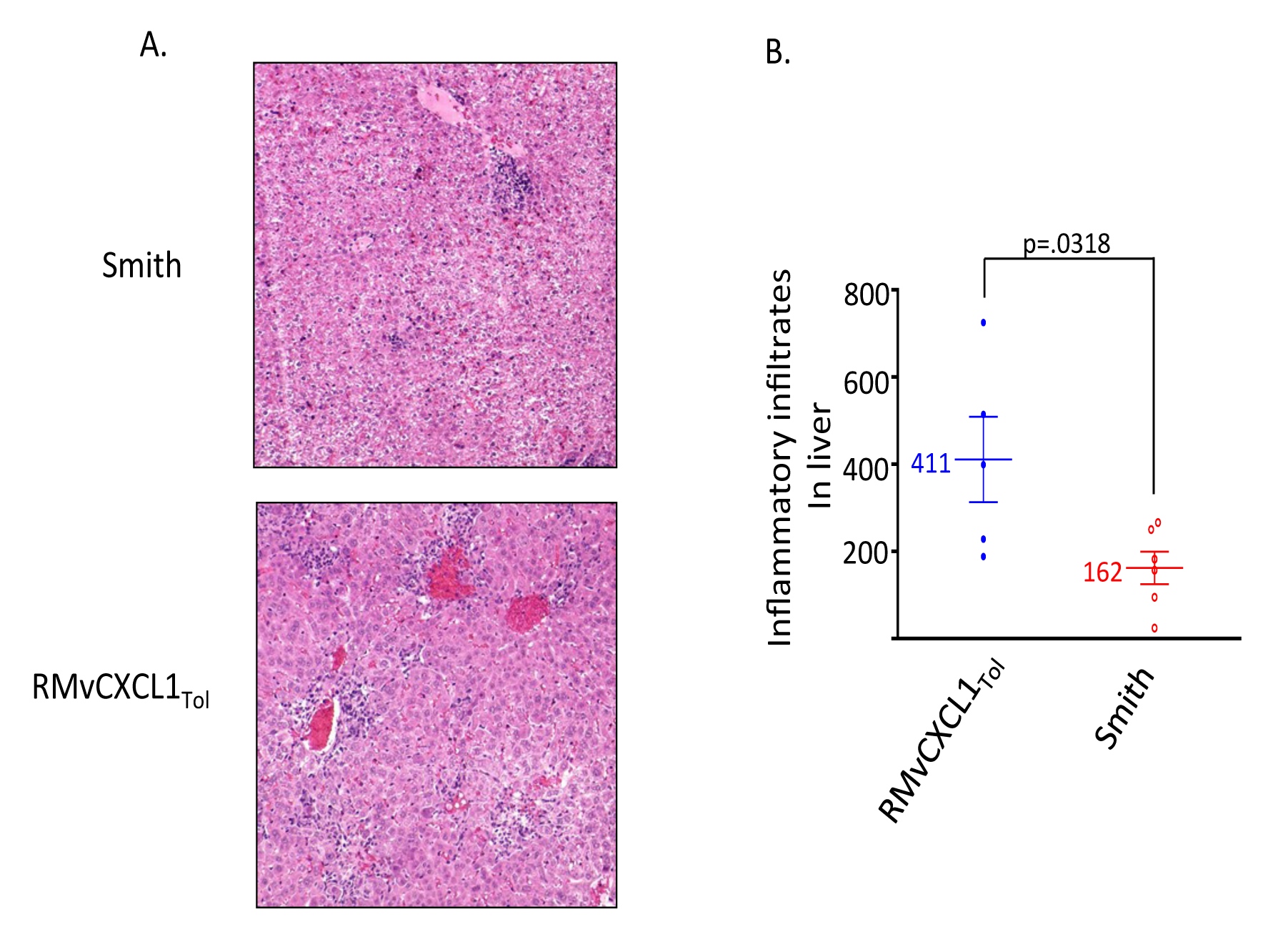

Supplement: FIG S5 [file mBio.01289-19-sf005.tif]
